# From the Editor's desk

**DOI:** 10.1155/S1463924679000201

**Published:** 1979

**Authors:** Peter B. Stockwell


### Commentary

### Why automation lags

The need for the creation of the *Journal of Automatic Chemistry* — as Peter Stockwell has implied in his commentary for the first issue — has arisen from the interdisciplinary nature of the field of automation. Thus, in contrast to other new journals which are dedicated to narrower and more specialised topics we are encompassing a variety of fields each of which knows their own dedicated literature.

In lectures and papers it has been pointed out again and again that automation is a systems problem. The necessity for a combination of a variety of disciplines represents a conceptual barrier for automation to spread to new areas. It is particularly true for analytical chemistry where much of the work is related to the *person* of the analytical chemist and his touch and intuition for the situation. This fact certainly has contributed to a certain lag in automation of especially industrial analytical chemistry. It is interesting to note that a similar situation may be observed in office automation where increases in efficiency through automation were successfully prevented for a long period of time with the argument that "my work cannot be automated because of its personal nature".

But accepting the fact that automation is interdisciplinary is not enough. Care has to be taken that all aspects are included in any project of automation. This concerns also organizational and psychological aspects that arise with automation. It is well known that the quantitative benefits of automation ensue from an increase in productivity. In many cases it is implied that a change in the organizational structure is a necessity and that required manpower is reduced. Although it is a widely accepted fact that automation is beneficial and has furthered our standard of living Trade Unions tend not to agree, they clearly see that in many cases their jobs are endangered.

It is not easy to point out to someone who is afraid of being made redundant that automation is a systems problem and that it will be good for the economy, our standard of living etc. Careful planning is required in all cases — for small and large projects — in which way automation is to be introduced to the personnel of an organization and in which way psychological and emotional reactions should be anticipated.

Failure to do so can become very costly and might be a major reason for automation to lag. There are examples abound in other disciplines where Unions have successfully resisted, and in many cases rightly so.

Nevertheless, the Unions should be aware that they cannot over any extended period of time hinder progress in the form of automation. During the industrial revolution of the last century obstruction to mechanization was a wide spread thing in several countries. Today one hardly thinks of it. The question today is only whether automation will happen together or in conflict.

It is desirable that papers published in the *Journal of Automatic Chemistry* will devote some space to this aspect. Whether automation will lag or not is ultimately decided whether or not automation can be made suitably acceptable to all concerned. And if this journal can help this purpose it will achieve a noble goal.

*R. W. Arndt*

### From the Editor's desk

By now the first issue of the Journal of Automatic Chemistry will have reached your desk and it is hoped that it fills a void in the literature and meets its aim of communicating the many facets of automation in the laboratory successfully. Its ultimate success depends of course on a ready input of technical papers and also on receiving new views and comments from the readership in general. The many comments which we have received generally praise the first issue for containing many interesting items. There has been some limited critisism of the balance between a clinical and industrial interest both within the issue itself and in the make up of the editorial board. Prof. Haeckel writes from Hanover *many thanks for the first issue of JAC (by the way, what is the official abbreviation?) which I think is a good start. The list of corresponding editors has been extended however, the medical laboratory is still over represented. Although I feel happy about this, it could mean that the new journal may become a Journal of Automation in Clinical Chemistry.*

On the first point the current trend and one which we support is for the full title of a journal to be maintained in literature references. An abbreviation to JAC certainly is confusing and other variations apart from *J. Automatic Chemistry* are untidy. The latter would seem therefore to be a suitable abbreviation if indeed one is necessary.

The balance between clinical industrial and various other facets of automatic analysis is one of course that is of much more concern. Ideally, it is not the aim of this journal to discuss the various application areas but rather the aspects which directly relate to automation; in many cases these can be applied across disciplines. That is, to provide a forum for the cross-fertilization of ideas concepts and thoughts on automation. The aims and objectives of clinical and industrial applications are indeed different but there is no disguising that each can learn from the other. In clinical applications it can be argued that the instrumentation was developed well before the clear definition of the objectives and needs for clinical analysers were well thought out; or indeed before the implications of the generation of such vast amounts of data had been realised. In many industrial areas, automation is in an embrionic state, but with experiences gained in the clinical area, the correct level of automation and the successful implementation of it can be achieved. The additional market place generated in the industrial area will then hopefully encourage the commercial instrument companies to respond to the challenges and rethink the whole philosphy to both clinical and industrial automation.

Reflecting on the balances of editorial content and indeed the editorial board composition. The articles in the first issue cover reviews, technical papers and notes, notes on evaluation protocols, and reports on evaluations. These cover both clinical and industrial aspects equally. This issue continues to maintain the balance, but if more aspects of clinical chemistry remain in the fore in future this only relects a real world situation. Table I illustrates in a simple fashion the interests and experience of the various members of the editorial board. In the academic area particularly in the USA the interest of the board spans both clinical and industrial situations and this is illustrated. Recently three corresponding editors have been added. These are Mr. Christian Collombel, Hospital de Charpennes, Lyon, France, a clinical chemist; Dr. Bo Karlberg of Astra Pharmaceuticals, Sodertalje, Sweden, who has industrial experience of both continuous and flow injection principles; and Professor M. Bonner Denton, from the Chemistry Department of Arizona University, Tucson, USA, who, apart from teaching chemistry, has automation interests in both clinical and industrial applications. Further additions to the editorial board will continue in this manner to fully represent various national interests in an international journal dealing with both clinical and industrial facets.

One of the major events that have taken place since the last issue of the journal was the 8th Technicon International Congress, held at Wembley UK from 12-14 December, 1978. Whilst this is reported in this issue it is perhaps pertinent here to comment on both the size and scope of the meeting. Over 2000 people attended throughout the three days and this fact coupled with the range of lectures aptly shows the impact that Technicon have made on the automation scene since the concept of continuous flow was introduced over 21 years ago. Many of the products shown at the associated exhibitions were exhibited for the first time in the UK. The fast-HPLC system, represents a considerable advance in the thinking of commercial companies. Automation in chromatography both gas and liquid has long been misconstrued as being simply mechanical sample injection coupled to electronic data acquisition and reduction. The system designed by Technicon attempts to cover the full analytical problem from sample handling and pretreatment through to measurement and data reduction. The solution is however, a complex instrument which by its nature is a combination of both automatic chemistry and chromatography. This highlights one of the perenial problems associated with automation in general; that is, who operates the instrument and to whom is it marketed? The operation of the instrument may well be within the compass of a junior analyst but the successful long term operation of the instrument will demand the presence of a more highly skilled scientist. This specialist must have an indepth understanding of the system, be able to detect and correct faulty operation and be able to undertake modifications and adjustments to meet new circumstances, such as significant changes in sample composition, and to meet unusual situations. In the fast-HPLC system the need is to both understand the implications of the sample pretreatment system and the chromatography involved. The instrument is an automatic system and not an adjunct to a chromatograph. Incorrect control of the first stages will negate the usefulness of the chromatographic measurements. In vitamin analysis for example it is very easy to produce incorrect results, if one or other of the vitamins being analysed is unstable to the chemical environment. However, these problems should not distract from the value of such degrees of automation since these are the correct way to overcome many of the bottlenecks in the laboratory. It is vital that the implications of their use are recognised and the proper degree of training provided. Regarding the system as a fast-HPLC system and not as an automatic instrument system does not seem to be the most satisfactory approach.

The aim of this journal is to improve the understanding of the philosophy of automation and from time to time focus on training and education problems. In this respect the UK Chemical Society is sponsoring a summer school on Automatic Analysis. This and Analysis 1979, sponsored by this journal, provide two additional vehicles to improve the communications in the area.

The journal will flourish best given a lively interaction with the readership. Hopefully the content of this and future issues will encourage you to write your views and comments to us. We look forward to a full postbag in 1979.

*Peter B. Stockwell*

| Name | Country | Work Experience | | | | | Discipline of Application | | | | |
|---|---|---|---|---|---|---|---|---|---|---|---|
| | | Academic | Research Institute | Government | Commercial | | Clinical Chem | Process Control | Industrial Application | Pharmaceutical | Clin/Indust |
| Stockwell | UK | | | × | | | | | × | | |
| Arndt | Switz | | | | × | | | | × | | |
| Malmstadt | USA | × | | | | | | | | | × |
| Mitchell | UK | | × | | | | × | | | | |
| Burtiss | USA | | × | | | | × | | | | |
| Fearn | UK | | | | × | | | | × | | |
| Greary | Aust | | × | | | | × | | | | |
| Haeckel | Germ | × | | | | | × | | | | |
| Kenyhertz | USA | | | | × | | | | × | | |
| Okuda | Japan | × | | | | | × | | | | |
| Nadean | USA | | | | × | | × | | | | |
| Pardue | USA | × | | | | | | | | | × |
| Porter | UK | | | × | | | | | × | | |
| Roberts | UK | | × | | | | × | | | | |
| Simon | Switz | × | | | | | | | × | | |
| Squirell | UK | | | | × | | | | × | | |
| Stewart | USA | | | × | | | | | × | | |
| Werder | Switz | | | | × | | | | × | | |

### Notes for Contributors

### Presentation of manuscripts

Manuscripts should be typed (double-spaced) on one side of the paper only and with generous margins. The title should be brief and informative avoiding the word "new" and its synonyms. The full list of authors with their affiliations and full address(es) should appear on the title page. On a separate sheet an abstract of no more than 150 words is required. This should succinctly describe the scope of the contribution and highlight significant findings or innovations. It should be written in a style which can easily be translated into French and German.

*The Concise Oxford Dictionary* and Fowler's *Modern English Usage* (both published by Oxford University Press) should be used as the standard for spelling and grammar. Abbreviations should be limited to those generally recognised, or where a frequently occuring term is abbreviated it should, in the first instance, be explained thus "flow injection analysis (FIA) ..." and the abbreviation used thereafter. Abbreviations, for standard measures and units should follow SI recommendations. There are various publications giving guidance on the use of SI units.

References should be indicated in the text by numerals following the author's name, i.e. Skeggs [6]. On a separate sheet of paper, list all references in numerical order thus:

[6] Skeggs, L.T., *American Journal of Clinical Pathology,* 1959, **28**, 311.

Note that journal titles are given in full. Where there is more than one author, the form Foreman et al. should be used in the text but all authors should be named in the list of references. When reference is made to a chapter in a book the reference should take the following form:

[7] Malmstadt, H.V. in "Topics in Automatic Chemistry" Ed. Stockwell P.B. and Foreman J.K. 1978 Horwood, Chichester, pp. 68-70.

Only work which has been published or has been accepted for publication should be cited. Avoid the citation of documents which are subject to restricted circulation, patent literature, unpublished work and personal communications. The latter can be mentioned in the text in parenthesis.

To illustrate a paper line diagrams are preferred to photographs. Photographs should only be used when they significantly add to the discussion. Diagrams, charts and graphs should be carefully drawn in black ink on stout card or heavy quality tracing paper. Most illustrations are reduced for publication; to allow for this originals should be between 16 and 36 cm wide (the depth must not exceed 50 cm). The lettering of diagrams should be sufficiently clear to withstand reduction. Except in the case of proper names, all lettering should be in lower case print. If photographs are used they must be supplied in the form of clear, unmounted, glossy, black and white prints. "Instant" photographs are not normally acceptable. All illustrations must be identified on the reverse showing the figure number and the author's name.

Each illustration should have a fully explanitory caption. Captions should be typed together on a separate sheet of paper; they must *not* be an inseparable part of the illustration.

